# Methods for improving the thermal performance of thermal bridges of lightweight steel-framed buildings

**DOI:** 10.1371/journal.pone.0314634

**Published:** 2024-12-17

**Authors:** Xuejun Qi, Yuxin Tan, Jinsheng Tan, Xiaohong Li

**Affiliations:** 1 School of Architecture and Civil Engineering, Xihua University, Chengdu, China; 2 China 19th Metallurgical Group Corporation Limited, Chengdu, China; Southwest Jiaotong University, CHINA

## Abstract

The existence of a large number of thermal bridges in the Lightweight Steel-Framed (LSF) building leads to its energy loss. The reduction of the heat transfer of thermal bridges is crucial for increasing the thermal performance of the building envelope. In this study, the infrared technology was first used to measure the temperature of LSF buildings, and clear thermal bridge junctions were determined. The THERM software was then used to simulate the thermal bridge of the external wall-beam junction, external and internal corners of the external wall, and cornice. Finally, according to the results obtained by numerical simulation, the thermal bridge was improved for reducing its thermal loss and meeting the design standard of thermal bridge free. The results of the simulation showed that, when the thickness of the rock wool (RW) of the external wall is greater than 75 mm, the linear thermal bridge coefficient (Ψ-value) at the junction of the external wall-beam is less than 0.01 W/(m·K), which meets the design standard of thermal bridge free. When polyurethane (PU) is used instead of RW for the external wall, its external corner meets the design standard of thermal bridge free in the case where the thickness of the PU is greater than 65 mm. The internal corner of the external wall can meet the design standard of thermal bridge free when PU is used instead of RW. The thermal bridge of cornice can meet this standard by adding a PU thermal insulation layer at the indoor sides, having a thickness greater than 20 mm. Studying the thermal bridge of LSF buildings allows to promote the development of the green building technology in China.

## 1. Introduction

Due to the fast development of urbanization, the energy consumption used for building construction and operations is rapidly increasing. During 2023, the building sector accounted for 38.2% of the total carbon emission in China [1]. The lightweight steel-framed (LSF) building is suitable for construction in many geological conditions of the foundation and 5 floors below the building. It can significantly reduce the foundation construction cost, and it is less affected by the weather in the construction process. In addition, the LSF buildings have many advantages, including the small weight with high mechanical strength, high architectural flexibility for retrofitting purposes, high mechanical strength, suitability for prefabrication, cost-effectiveness, and safety at work [2, 3]. Cold-formed steel is the main structural material in LSF buildings [4–6]. It is usually used to form thermal bridges due to its high thermal conductivity. In some conditions, the thermal bridges may affect the thermal comfort of the house, and even damage its steel structure. In fact, the thermal bridges account for 23% of the total transmission heat loss of a building envelope [7]. Hence, in the latter, they play a crucial role in the energy saving of the LSF buildings.

When a thermal bridge is present, the surface temperature of the steel will decrease, which may lead to condensation, mold, and other related issues [8]. In addition, the thermal bridge decreases the thermal resistance of the wall and increases the heat transfer of the building envelope [9, 10]. Existing studies showed that the thermal bridges account for approximately 40% of the total annual energy in Spain [11]. Zhao et al. [12] showed that the annual heating and cooling loads of thermal bridges account for 1.7–8.2% of the total annual heating and cooling loads. De Angelis et al. [13] studied the double-layer cold-formed thin-wall light steel prefabricated building filled with mineral wool. They deduced that in general, the heat transfer coefficient of the wall is low without considering the LSF, and the difference is as high as 74%. Zalewski et al. [14] studied the thermal bridges in prefabricated building walls. They deduced that the heat losses in front of the steel frame are two times much important as elsewhere. Ge et al. [15] studied the dynamic impact of thermal bridges on the energy consumption of residential buildings in Canada. Their results showed that, in hot climate, the presence of thermal bridges increases the annual cooling load by 20%. In addition, for four cities in Canada, the thermal bridges increase the annual space heating energy demand by 38–42%, and decrease the annual space cooling energy demand by 8–26% [16, 17]. Song et al. [18] studied the linear and point thermal bridges in steel truss metal panel curtain wall systems. They demonstrated that during the night in winter, the average external surface temperature at the thermal bridge is 3.1–4.9°C higher than that at the center of the metal panel.

O’Grady [19] studied the impact of the wind velocity on the Ψ-value of thermal bridges. His results showed that the Ψ-value highly depends on the wind velocity. In addition, he adopted the infrared thermography technique (ITT) to study the heat loss of thermal bridges in buildings. His results showed that the difference between multiple thermal bridging heat losses q_TB_ and those measured in calibrated hot box tests is in the range of −5–2.5%, while that between the Ψ-values obtained from the ITT and those measured in calibrated hot box tests is in the range of 1–7% [20, 21]. Mao et al. [22] studied the thermal bridge effect of vacuum insulation panels. They deduced that the thermal bridge increases with the increase of the mental foil thickness, and exhibits higher heat losses when decreasing the thickness of the vacuum insulation panel. Milovanović [23] showed that the impact of thermal bridges on the heating energy demand is significant while that on the cooling energy is less pronounced.

Kim et al. [24] proposed a method for the detection of linear thermal bridges from thermal images, using image processing techniques and artificial neural networks. Romero et al. [25] proposed two novel construction improvements to reduce the impact of the thermal bridges on the energy demand in three Spanish cities. Their results showed that these improvements reduce the linear thermal transmittance of the thermal bridge and the heating demand to the ranges of 20–63.4% and 16.1–22.6%, respectively. Zhang et al. [26] used thermal break elements to increase the thermal resistance of a balcony thermal bridge in South Korea. Their results showed that the linear thermal transmittance of the thermal bridges with thermal break elements is reduced by more than 60%, and the heating energy demands are reduced by more than 8%. Real [27] demonstrated that structural lightweight aggregate concrete can be used instead of normal weight concrete to reduce the thermal bridging effects in buildings. Miguel et al. [28] studied a cost-effective solar panel used as a building wall. Their results showed that the back pass solar air collector is able to reduce the heating load of the test buildings by approximately 20%.

Many differences exist between the LSF buildings and the traditional ones, mostly in terms of design. However, the studies on the thermal bridges of LSF buildings and the heat transfer performance of thermal insulation materials are still few. Therefore, this paper aims at studying the heat transfer characteristic of typical thermal bridges, such as the external wall-beam junction, external corner of the external wall, internal corner of the external wall, and cornice. Improvement measures for thermal bridges are then proposed. The obtained results provide a reference for the future design of thermal bridge free, which is of great significance for the energy conservation of LSF building envelope.

## 2. Methodology

### 2.1 Structure of thermal bridges

**Fig 1:**
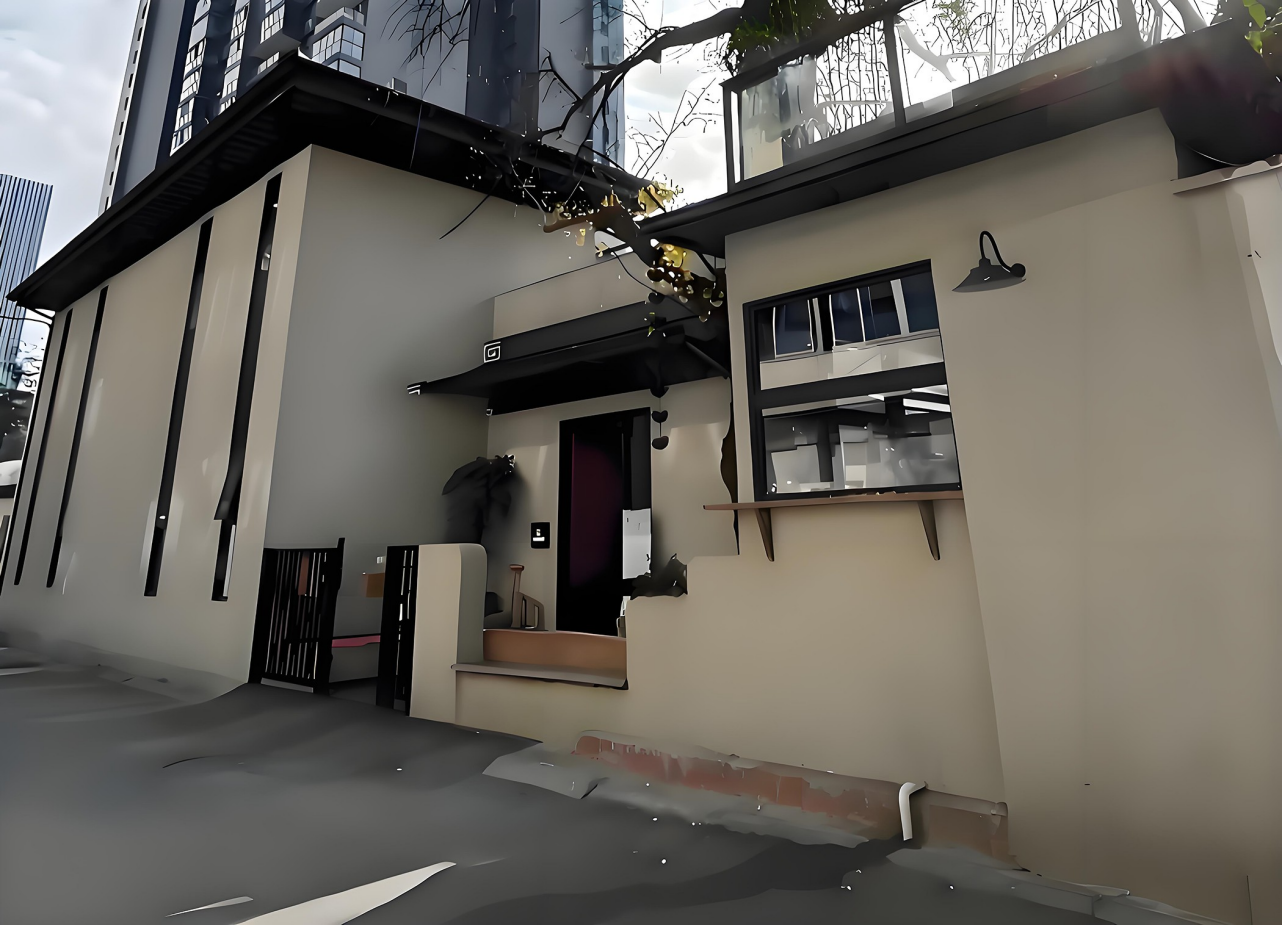
LSF building.

**Fig 2:**
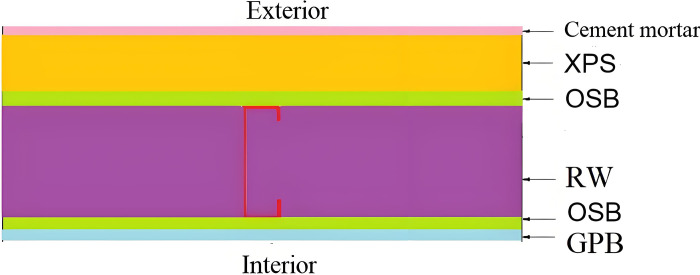
Structure of the exterior wall.

The LSF building adopts cold-formed steel, which has very high seismic performance and can significantly reduce the impact of natural disasters, such as earthquakes, on the building. Therefore, the proportion of LSF buildings in new ones in Chengdu continues to increase. Therefore, an LSF building in Chengdu (China) is considered as the object of this study (Fig 1). RW is often used as thermal insulation material for building envelope. The structure of the exterior wall of this LSF building is shown in Fig 2. It can be seen that the exterior wall is made of cement mortar, and extruded polystyrene (XPS) is used as external insulation material, covering the oriented strand board (OSB). In addition, the LSF is made of galvanized cold-formed thin-wall C-type steel with 600 mm spacing. Table 1 presents the parameters of the exterior wall of the LSF.

**Table 1:** Parameters of the exterior wall of the LSF building.

Materials	Width (mm)	Thermal conductivity W/(m·K)	Reference
Cement mortar	20	0.930	[29]
Extruded polystyrene(XPS)	25	0.030	[30]
Oriented strand board (OSB)	10	0.120	[29]
Rock wool (RW)	140	0.036	[29]
C-type steel (C140×40×15×1.5)	140	58.200	[30]
Oriented strand board (OSB)	10	0.120	[29]
Gypsum board (GB)	9.5	0.330	[30]

**Fig 3:**
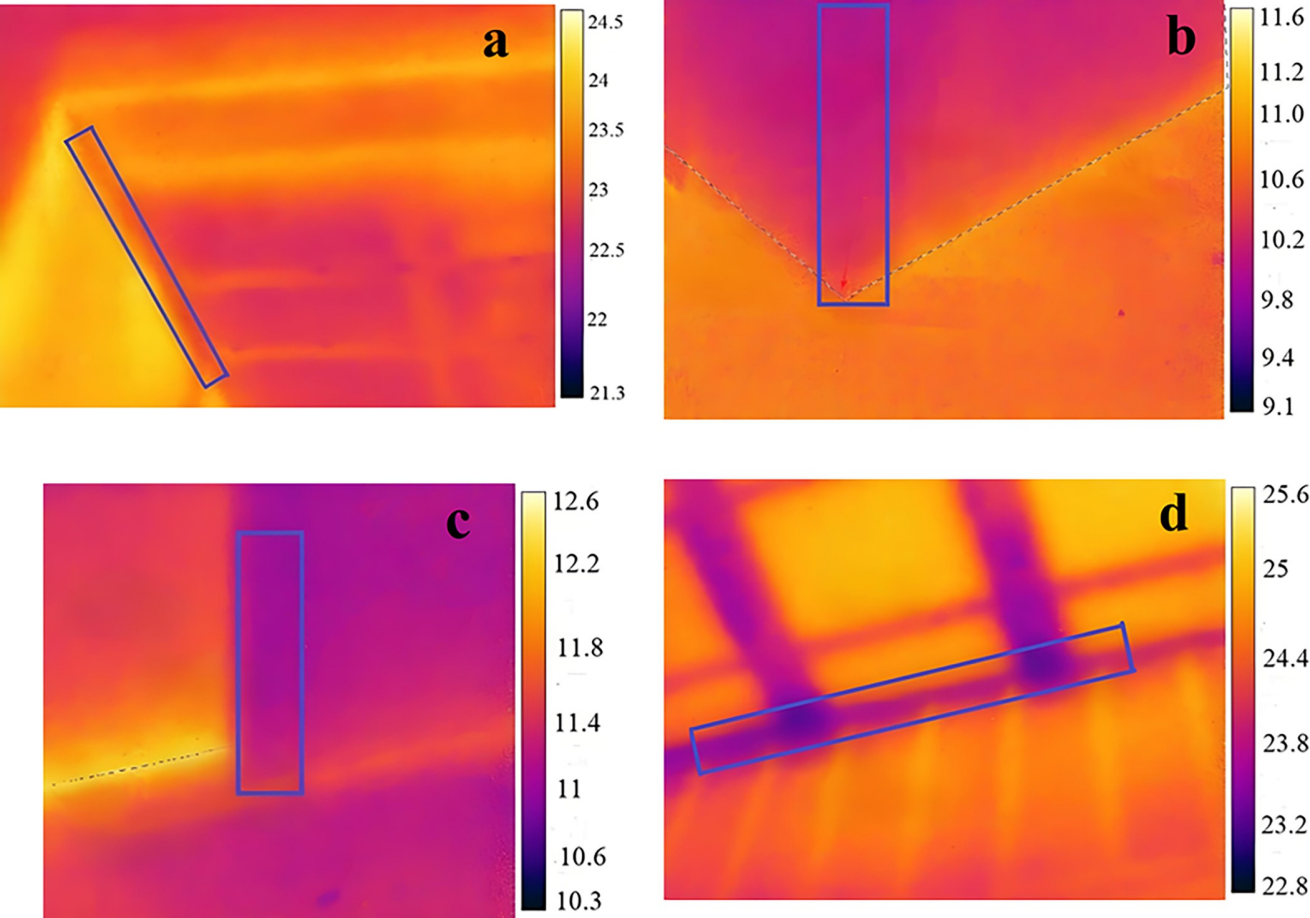
Thermal infrared image of thermal bridge. a. Exterior wall-beam, b. External corner, c. Internal corner, d. Cornice.

**Fig 4:**
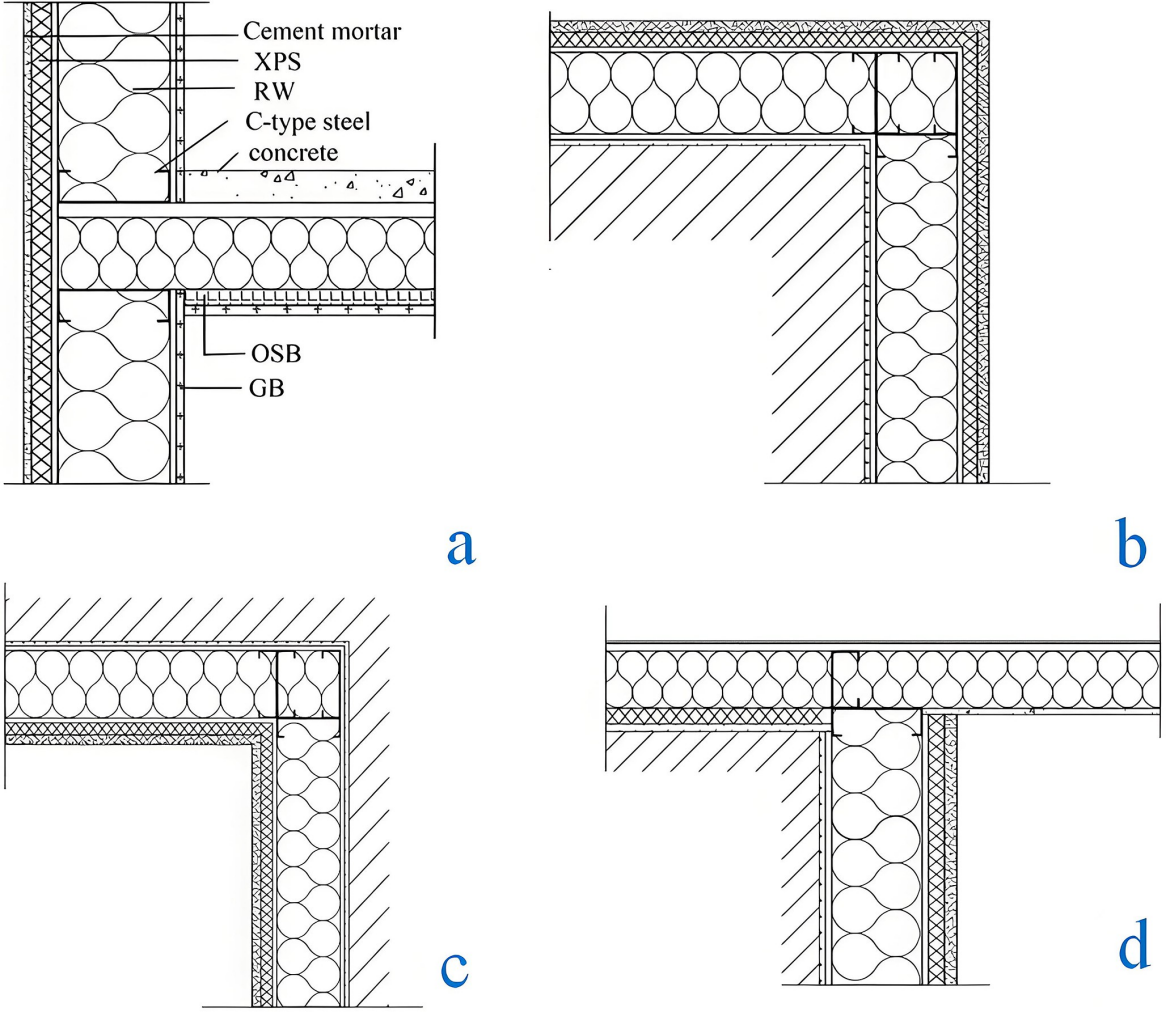
Structure of thermal bridge junctions. a. External wall-beam junction, b. External corner of the external wall, c. Internal corner of the external wall, d. Cornice.

Fig 3 shows a thermal infrared image of the junction of typical thermal bridges. It can be seen from Fig 3(A) that the temperature at the connection between the external wall and the beam is significantly lower than the surrounding temperature, which forms a thermal bridge phenomenon. It can be observed from Fig 3(B) and 3(C) that the temperature at the corner is lower than that at the surface of the external wall, which is due to the change in the area of the corner of the wall, leading to thermal bridge effects. Fig 3(D) shows the thermal bridge phenomenon at the cornice. The structure of the thermal bridge in the LSF buildings is shown in Fig 4.

### 2.2 Properties of the materials

The selection of building thermal insulation materials should consider the thermal insulation performance, fire performance, durability, and environmental protection. ‌Choosing the right thermal insulation material is crucial to the energy efficiency, safety and durability of a building. At present, RW, polystyrene, polyurethane (PU), expanded polystyrene (EPS), extruded polystyrene (XPS), and fiberglass are often used as thermal insulation materials in exterior walls [3, 31]. The thermal insulation materials can be divided into organic, inorganic, and composite insulation. In China, the organic thermal insulation materials used for building walls mainly include EPS, XPS, and PU. The inorganic thermal insulation materials used for building walls mainly include RW, glass wool, silicate, and foam glass. The organic materials are usually lighter than inorganic materials, and they can reduce the weight and foundation load of the building. This paper mainly studies the impacts of PU, EPS, and XPS on the heat transfer characteristics of thermal bridges. Table 2 presents the parameters of these materials.

**Table 2:** Parameters of thermal insulation materials.

Thermal insulation materials	Thermal Conductivity W/ (m·K)	Reference
Polyurethane (PU)	0.024	[30]
Extruded Polystyrene (XPS)	0.030	[29]
Expanded polystyrene (EPS)	0.038	[30]

### 2.3 Numerical simulation parameters setting

The THERM software, which is widely used to study the thermal performance of walls [27, 32], is used in this paper to study the heat transfer characteristics of thermal bridges. The boundary conditions were set as follows: surface heat transfer coefficients of inner and outer surfaces of 8.7 W/(m^2^·K) and 23.0 W/ (m^2^·K), respectively [30]. The other surfaces were set as adiabatic surfaces. This paper focuses on the heat transfer characteristics of thermal bridges in winter. In the conducted numerical simulation, it is assumed that the outdoor air temperature, indoor air temperature, and relative humidity of air are 3.8°C, 20°C, and 60%, respectively.

### 2.4 Calculation of linear heat transfer coefficient

The linear heat transfer coefficient (*Ψ*) is calculated using Eq (1) [30]. Eq (2) is then substituted into Eq (1) yielding Eq (3). Finally, Ψ can be calculated using Eq (5).

$$\Psi =\frac{Q^{2D}-KA(t_{i}-t_{e})}{l(t_{i}-t_{e})}=\frac{Q^{2D}}{l(t_{i}-t_{e})}-KC$$ (1)

where *Ψ* is the linear heat transfer coefficient (W/m∙K), *Q*^*2D*^ is the heat transfer through a building envelope containing a thermal bridge (W) (it can be determined by the THERM software), *K* is the heat transfer coefficient of building envelope (W/(m∙K)), *A* is the area of building envelope of Q^2D^ (m^2^), *t*_*i*_ and *t*_*e*_ are respectively the air temperatures at the interior and exterior sides of the building envelope (°C).

$$Q^{2D}=K^{2D}∙A(t_{i}-t_{e})$$ (2)

$$\Psi =\frac{K^{2D}∙A(t_{i}-t_{e})}{l(t_{i}-t_{e})}-KC$$ (3)

A=C∙l (4)

where *K*^*2D*^ is the two-dimensional average heat transfer coefficient of the building envelope with thermal bridge (W/(m^2^∙K)), *C* and *l* are respectively the width and length of the building envelope (m) (*l* is set to 1 in this paper) [30].

$$\Psi ={(K}^{2D}-K)C$$ (5)

In the simulation process, the influence range of the thermal bridge is 1.2–2 times that of the wall thickness [33]. Therefore, in this study, the influence range of the thermal bridge is set to 400 mm. Based on the design standard of passive house, the maximum Ψ-value should be less than 0.01 W/(m·K) [34]. Therefore, a Ψ-value less than 0.01 W/(m·K) is considered to meet the design standard of the thermal bridge free.

## 3. Results and discussion

### 3.1 Analysis and improvement of the thermal bridge of the external wall-beam junction

**Fig 5:**
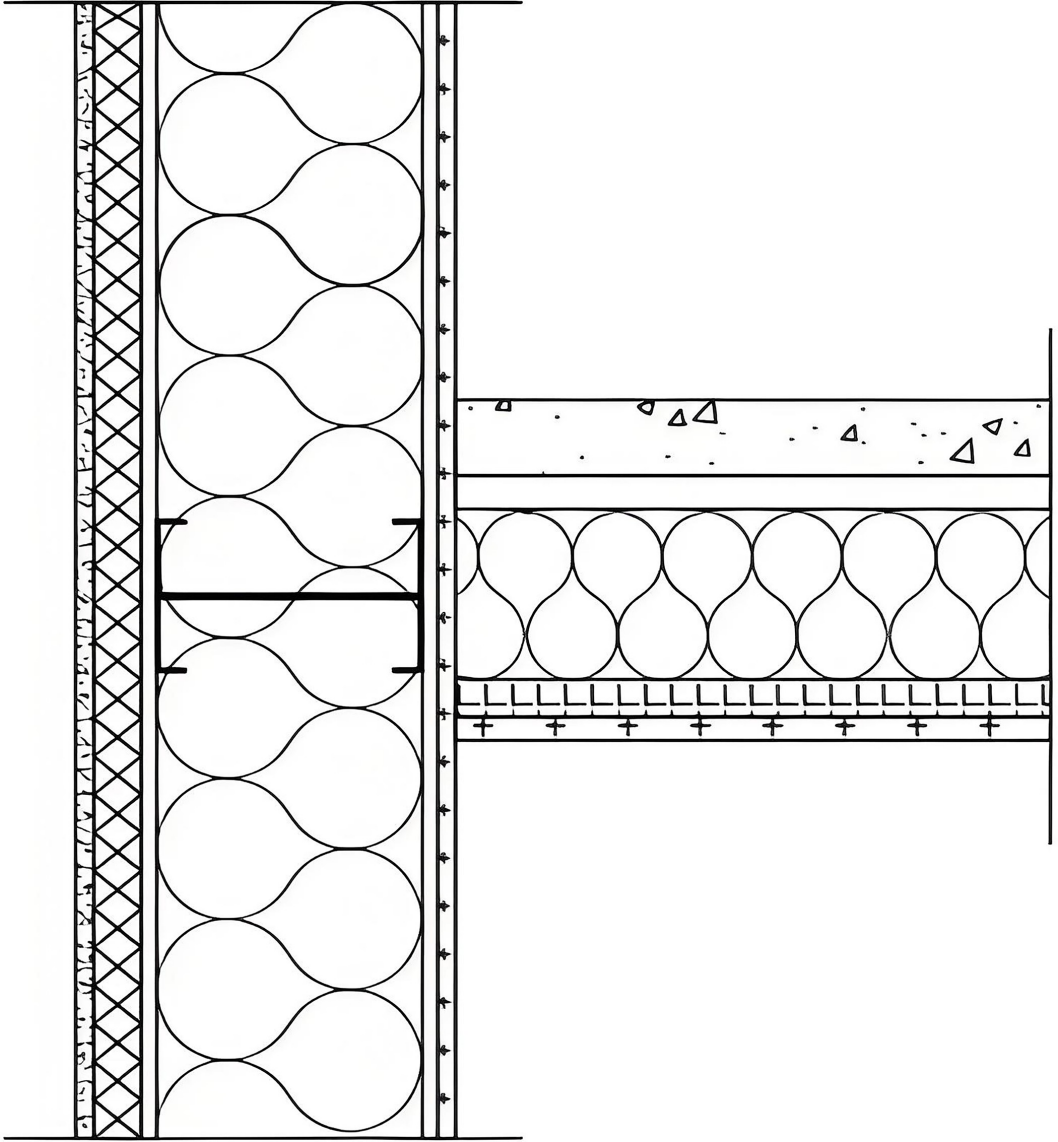
Improved structure of the exterior wall-beam junction.

The high thermal conductivity of the C-type steel, the different thermal conductivities of the thermal insulation materials, and the damage of their continuity are the main reasons for the thermal bridge of LSF buildings [35]. Hence, ensuring continuity is the first step in the improvement of the thermal bridge. Otherwise, the thermal bridges can undergo breaking treatment. The improvement method consists in connecting the prefabricated beams by external hanging and installing them by supporting anchorage. The improved structure is shown in Fig 5.

**Fig 6:**
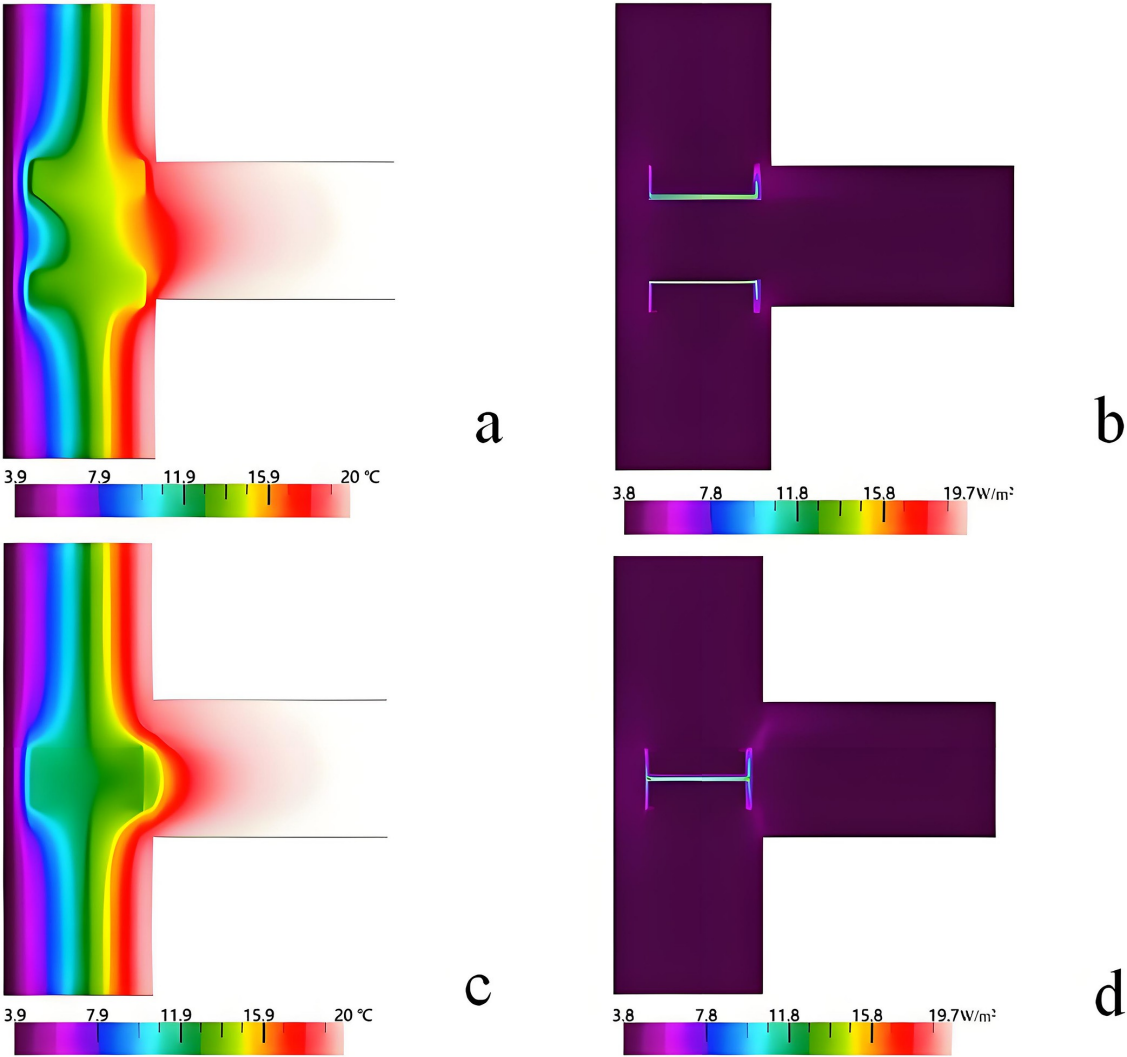
Comparison between the temperatures and heat flux distributions before and after improvement. a. Temperature distribution before improvement, b. Heat flux distribution before improvement. c. Temperature distribution after improvement d. Heat flux distribution after improvement.

Fig 6 shows the simulation results of the temperature and heat flux distribution before and after improvement. The calculated results of K^2D^ and Ψ are respectively 0.365 W/(m^2^·K) and 0.033 W/(m·K), which cannot meet the design standard of thermal bridge free. After improvement, K^2D^ and Ψ become equal to 0.287 W/(m^2^·K) and 0.019 W/(m·K), respectively. Since the Ψ-value is greater than 0.01W/(m·K), it can be concluded that simply changing the connection mode cannot reduce the thermal bridge effects.

**Fig 7:**
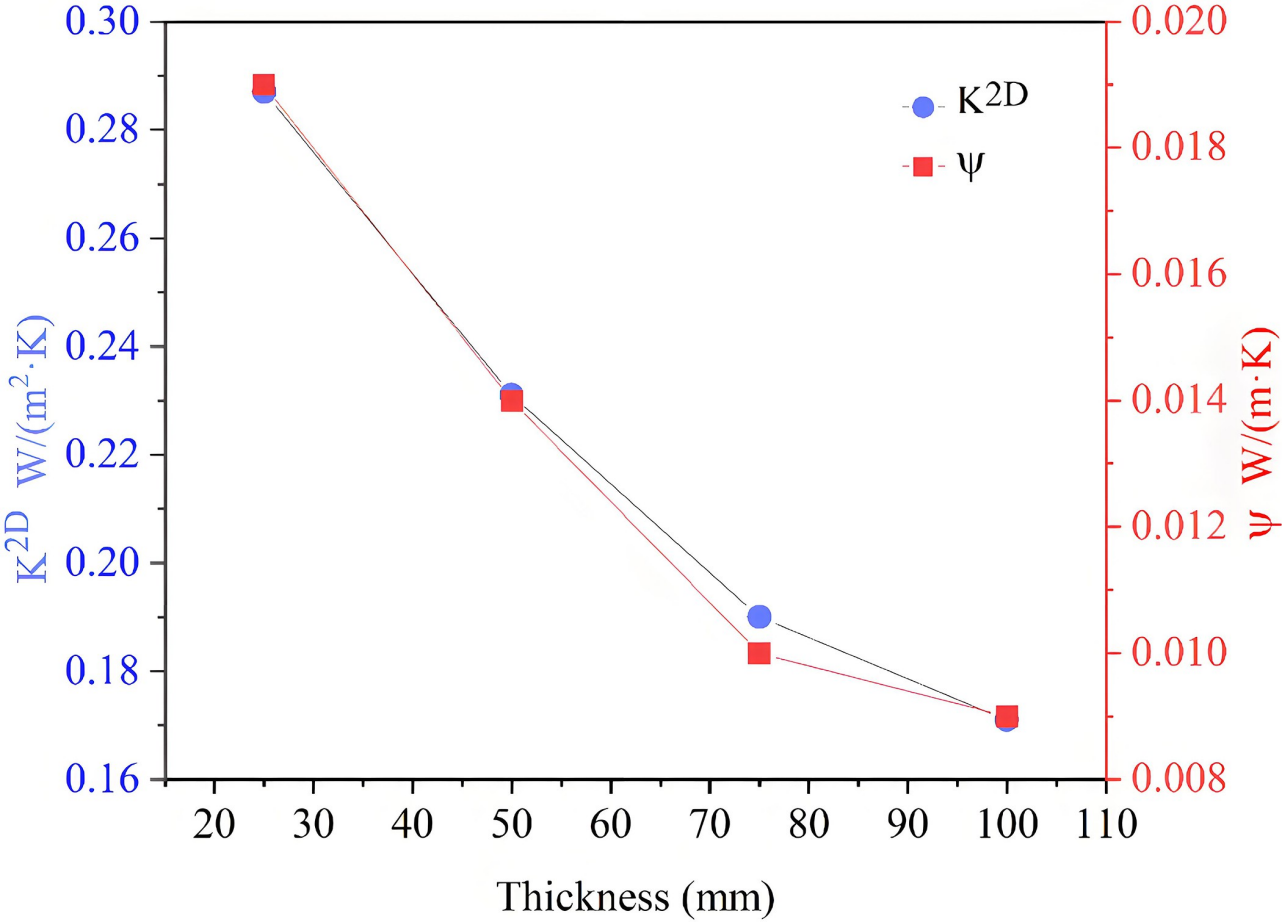
Impact of thickness on K^2D^ and Ψ.

The method which consists in increasing the thermal resistance of the thermal bridge can be used to deal with the thermal bridge [30]. Hence, changing the thickness of RW is adopted to reduce the thermal bridge. The impact of the thickness on K^2D^ and Ψ is shown in Fig 7. It can be seen that K^2D^ and Ψ gradually decrease with the increase of the thickness. When the thickness is 75 mm, the Ψ-value is 0.01 W/(m·K), which meets the design standard of thermal bridge free. This demonstrates that the increase of the thickness of RW can significantly reduce thermal bridge effects.

### 3.2 Analysis and improvement of the thermal bridge of the external corner

**Fig 8:**
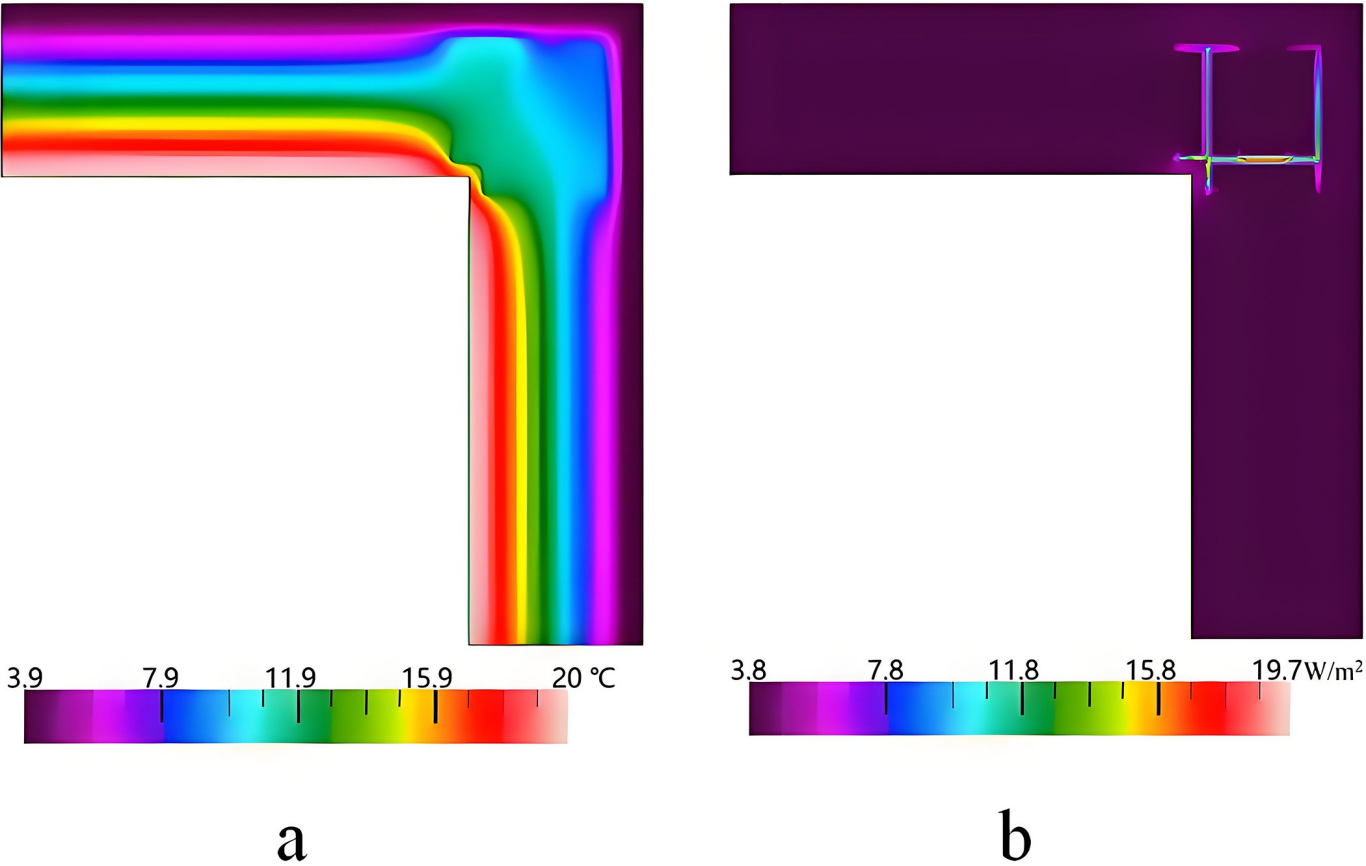
Simulation results of the external corner of the external wall. a. Temperature distribution, b. Heat flux distribution.

Fig 8 shows the simulation results of the thermal bridge of the external corner. It can be seen that the highest and lowest temperatures are 19.7°C and 3.9°C, respectively. The heat flux is concentrated in the part of the C-type steel. This is due to the fact that the high thermal conductivity of the steel causes thermal bridge effects. K^2D^ and Ψ are respectively 0.241 W/(m^2^·K) and 0.018 W/(m·K), which cannot meet the design standard of thermal bridge free.

**Fig 9:**
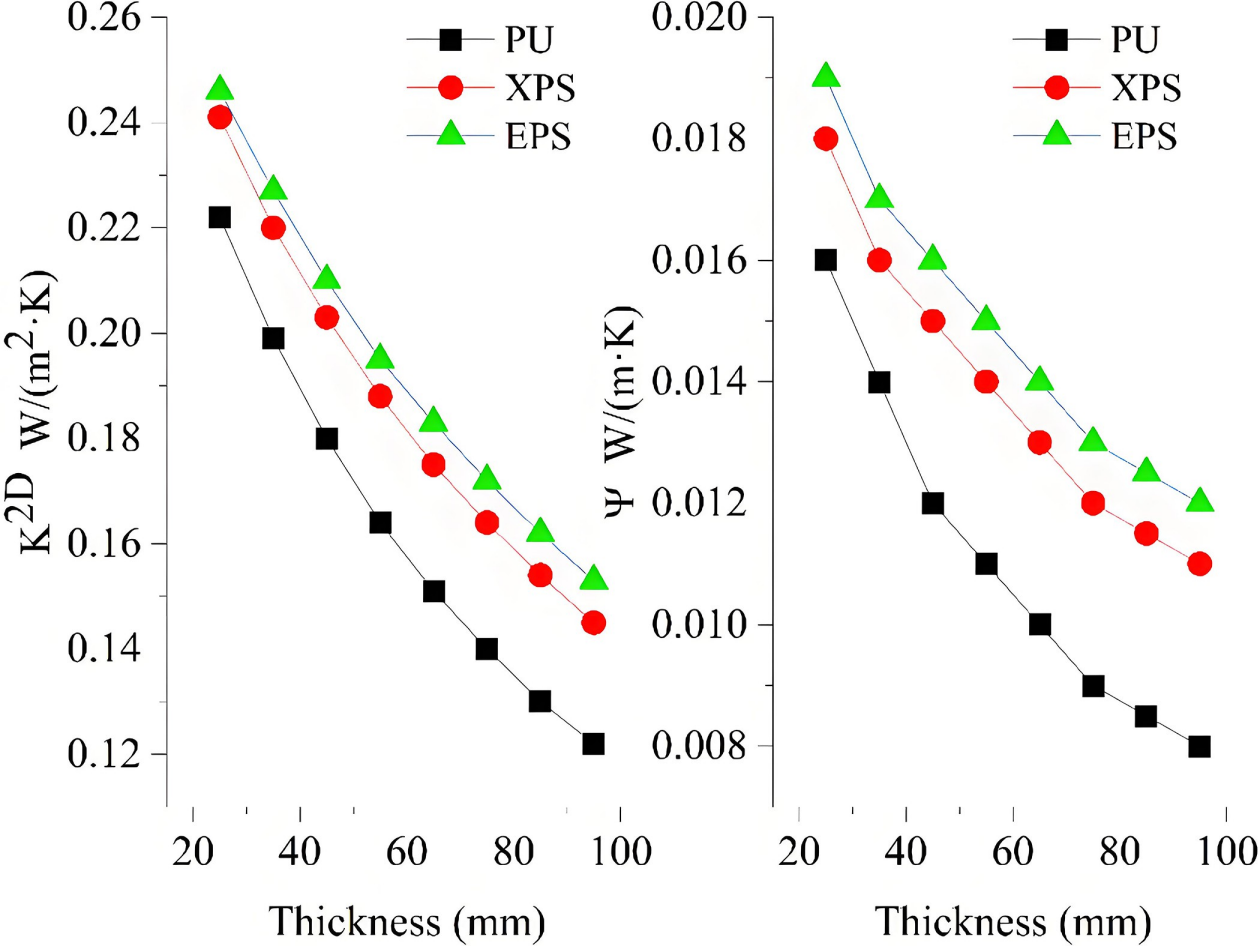
Impact of the thermal insulation layer thickness on K^2D^ and Ψ.

The thermal performance of the wall is affected by the thermal insulation material. Thus, the XPS, EPS, and PU are used as thermal insulation materials instead of the RW. Fig 9 shows the impact of the thermal insulation materials of different thicknesses on K^2D^ and Ψ. It can be seen that, when the thickness increases, all the values of K^2D^ and Ψ gradually decrease. When the thickness is 95 mm, the Ψ-values of the XPS and EPS are 0.011 W/(m·K) and 0.012 W/(m·K), respectively. It can be deduced from the aforementioned analysis that the XPS and EPS used as thermal insulation materials cannot satisfy the design standard of thermal bridge free for the underlying condition, even if the thickness reaches 105 mm. When the thickness increases to 65 mm, the Ψ-value of PU decreases to 0.010 W/(m·K), which meets the design standard of thermal bridge free. This is mainly because the thermal conductivity of PU is lower than EPS and XPS, which means that the sheet thickness required for PU is thinner when achieving the same insulation effect. Additionally, EPS and XPS are relatively water-absorbent, which can cause their performance to decline after prolonged use in humid environments. In contrast, PU is less absorbent and better able to maintain their insulation properties.

### 3.3 Analysis and improvement of the thermal bridge of the internal corner

**Fig 10:**
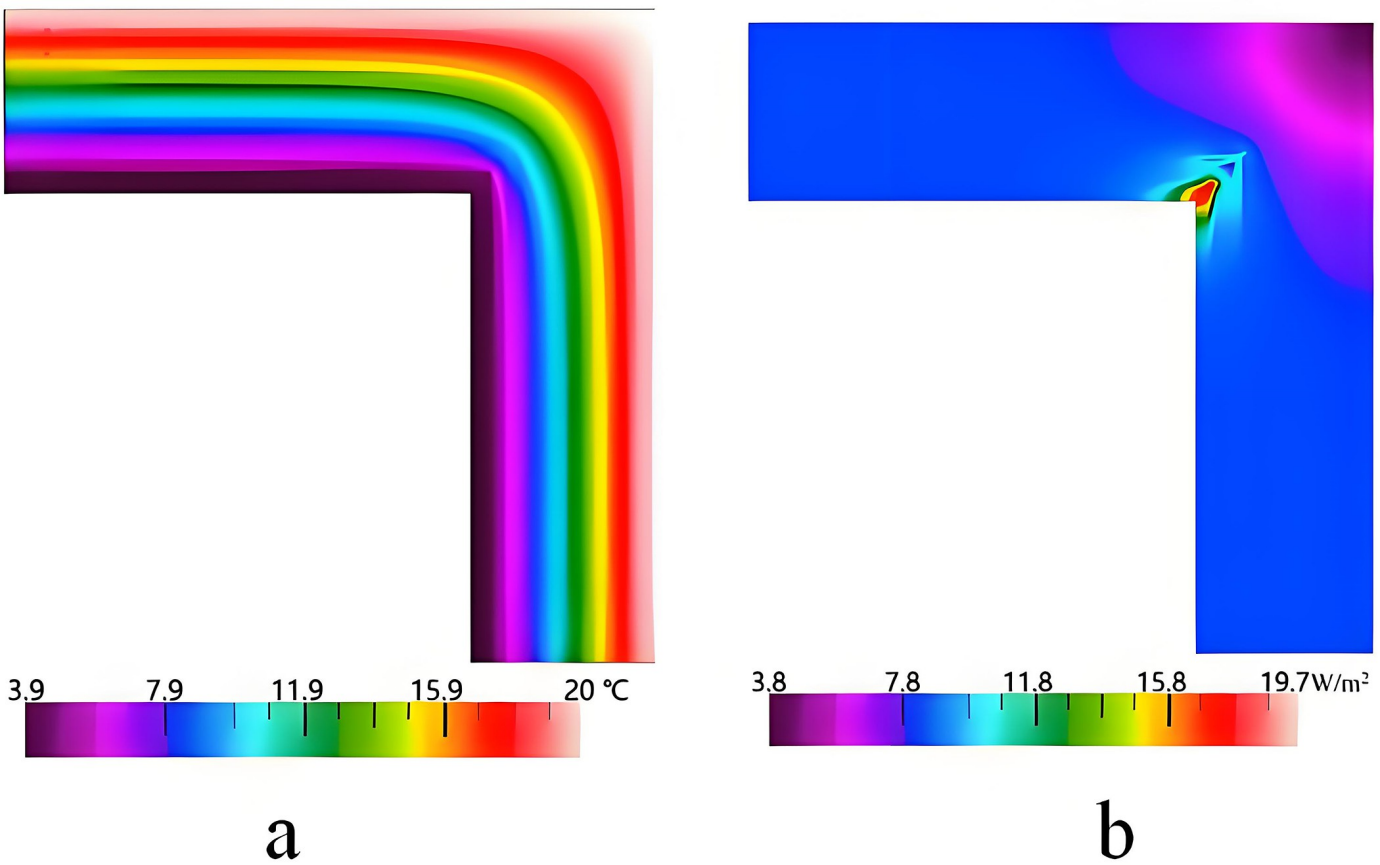
Results of the simulation of the internal corner of the external wall. a. Temperature distribution, b. Heat flux distribution.

Fig 10 shows the temperature distribution and heat flux distribution of the thermal bridge of the internal corner. It can be seen that the highest and lowest temperatures are respectively 19.9°C and 3.9°C, and the heat flux distribution is concentrated in the corner. The Ψ-value is 0.012W/(m·K), which demonstrates that it cannot meet the design standard of thermal bridge free.

**Fig 11:**
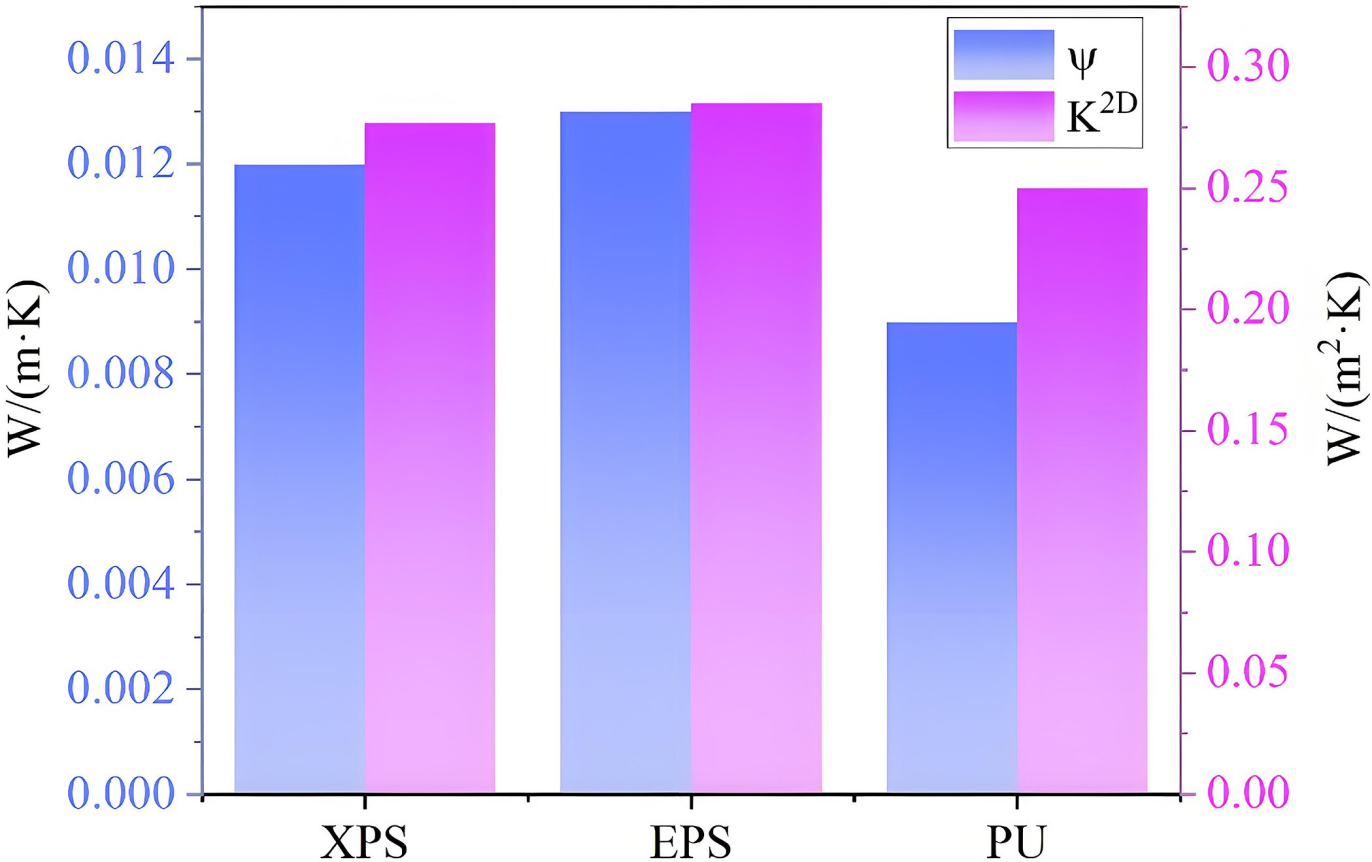
Impacts of different insulation materials on K^2D^ and Ψ.

Afterwards, the XPS, EPS, and PU are used as thermal insulation materials instead of the RW. The results of the simulation are shown in Fig 11. It can be seen that, when EPS and XPS are used as thermal insulation materials, the Ψ-value is greater than 0.01 W/(m·K). However, when PU is used as thermal insulation material, the Ψ-value is less than 0.01 W/(m·K). This demonstrates that the thermal bridge of the internal corner can meet the design standard of thermal bridge free by changing the thermal insulation material.

During the foaming process, a large number of tiny closed-cell structures are distributed in PU. When heat is conducted inside the material, it is necessary to overcome the resistance of gas molecules to transfer in the pores. In addition, the closed-cell structure effectively blocks the conduction flow of heat, which results in decreasing the heat conduction rate. The closed-cell structure can be considered as a barrier to heat, allowing to significantly reduces the heat loss and improve the insulation effect [36], in the case where PU has low thermal conductivity. This is consistent with the study presented in [30], which shows that the use of low thermal conductivity materials reduce the effect of the thermal bridge.

### 3.4 Analysis and improvement of the thermal bridge of the cornice

**Fig 12:**
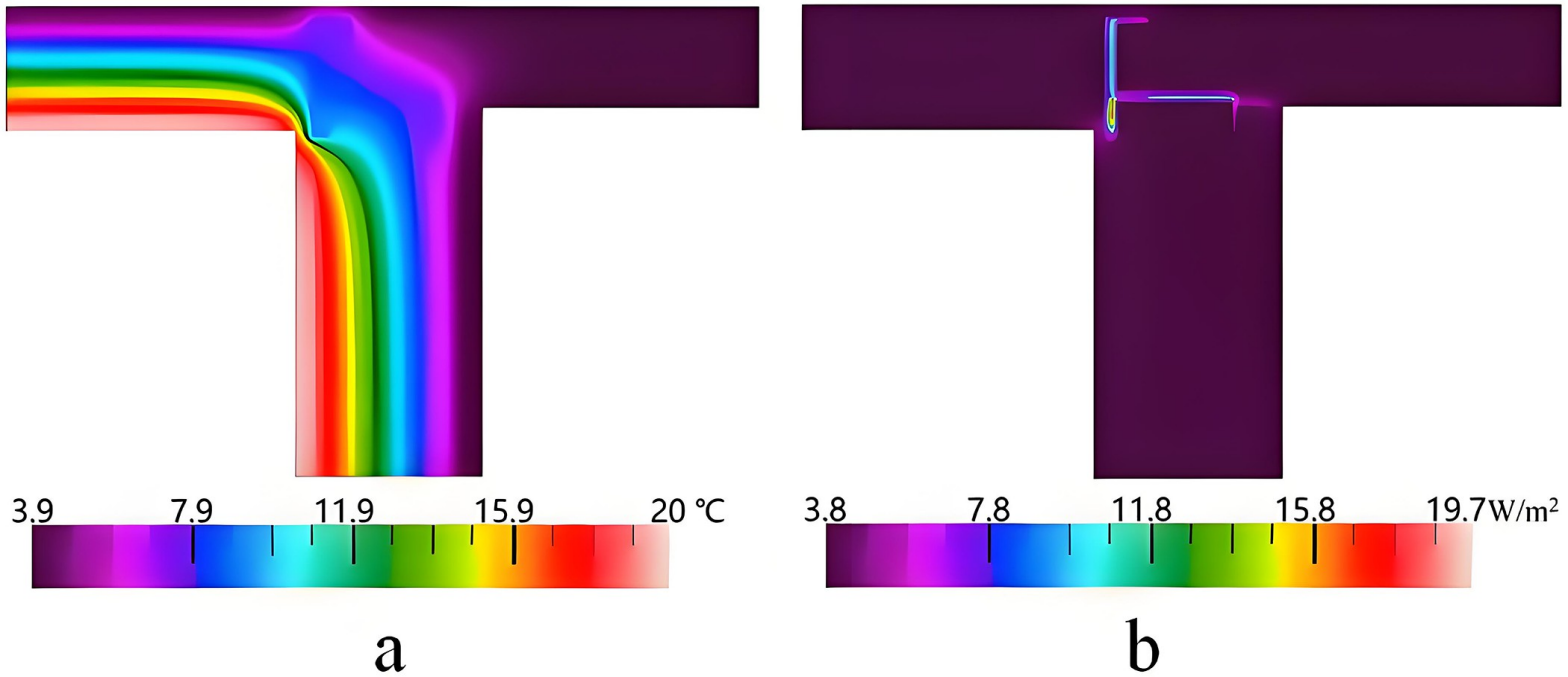
Simulation results of the cornice. a. Temperature distribution, b. Heat flux distribution.

Fig 12 shows the simulation results of the thermal bridge of the cornice. It can be seen that the highest and lowest temperatures are 19.74°C and 3.90°C, respectively. The heat flux at the cornice is concentrated at the connection part of the roof and the exterior wall. This is due to the fact that the discontinuity of the thermal insulation layer makes the heat transfer changes at the junction produce a thermal bridge.

**Fig 13:**
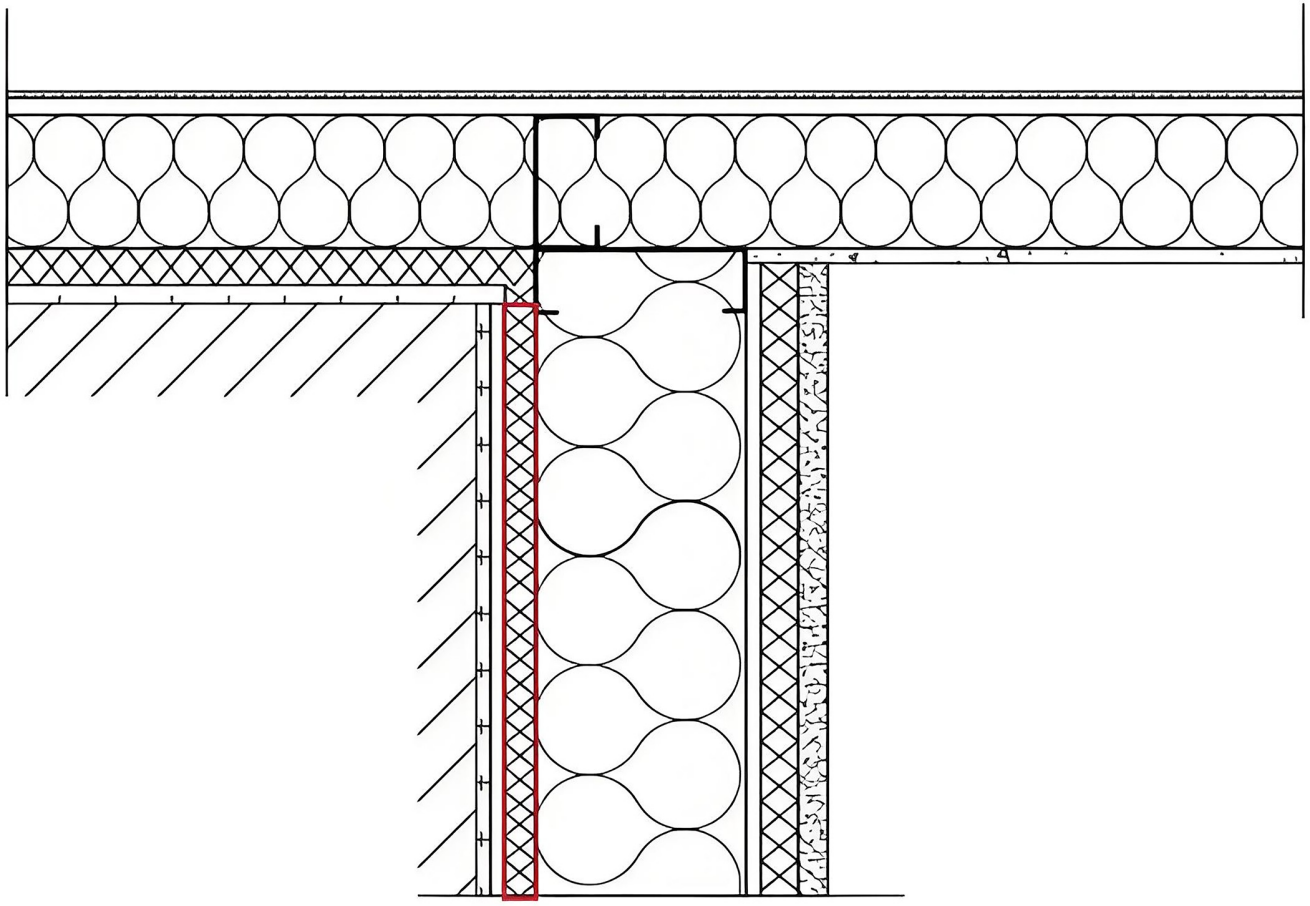
Cornice structure after improvement.

**Fig 14:**
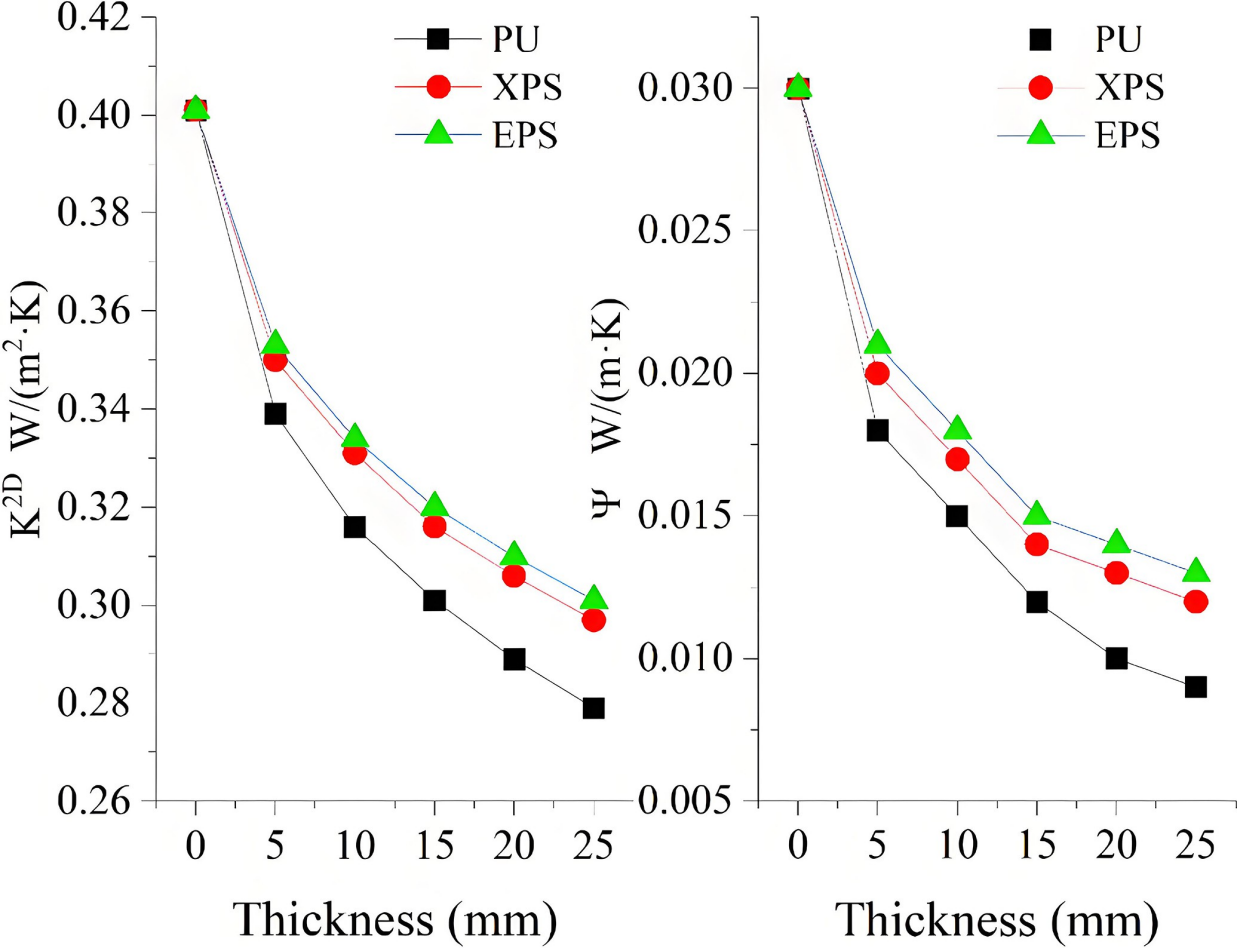
Impact of the thermal insulation layer thickness on K^2D^ and Ψ.

Based on the actual situation of the cornice, the improved method consists in the establishment of a wall thermal insulation layer with a roof thermal insulation layer form a continuous one, as shown in Fig 13. The impacts of the thicknesses on K^2D^ and Ψ are shown in Fig 14. It can be seen that K^2D^ and Ψ decrease with the increase of the thickness. When the thickness becomes greater than 10 mm, the decreasing trend starts to slow down. On the one hand, when the thickness is 25 mm, the Ψ-values of the XPS and EPS are 0.012 W/(m·K) and 0.013 W/(m·K), respectively. This does not meet the design standard of thermal bridge free. On the other hand, when the thickness of PU becomes greater than 20 mm, the Ψ-value is less than 0.010 W/(m·K), which meets the design standard of thermal bridge free. It can be concluded that the cornice thermal bridge free can be achieved when the thickness of PU is greater than 20 mm. This is consistent with the studies presented in [30] and [35], showing that the continuous thermal insulation materials can increase the thermal resistance and reduce the thermal loss of the thermal bridge. This is mainly due to the fact that the internal pores of the PU are independent, forming a continuous temperature insulation layer. In addition, the thermal conductivity of the PU is low, and thus the conduction rate of heat is decreased, which results in significantly reducing the energy loss of the building.

## 4. Engineering application

**Fig 15:**
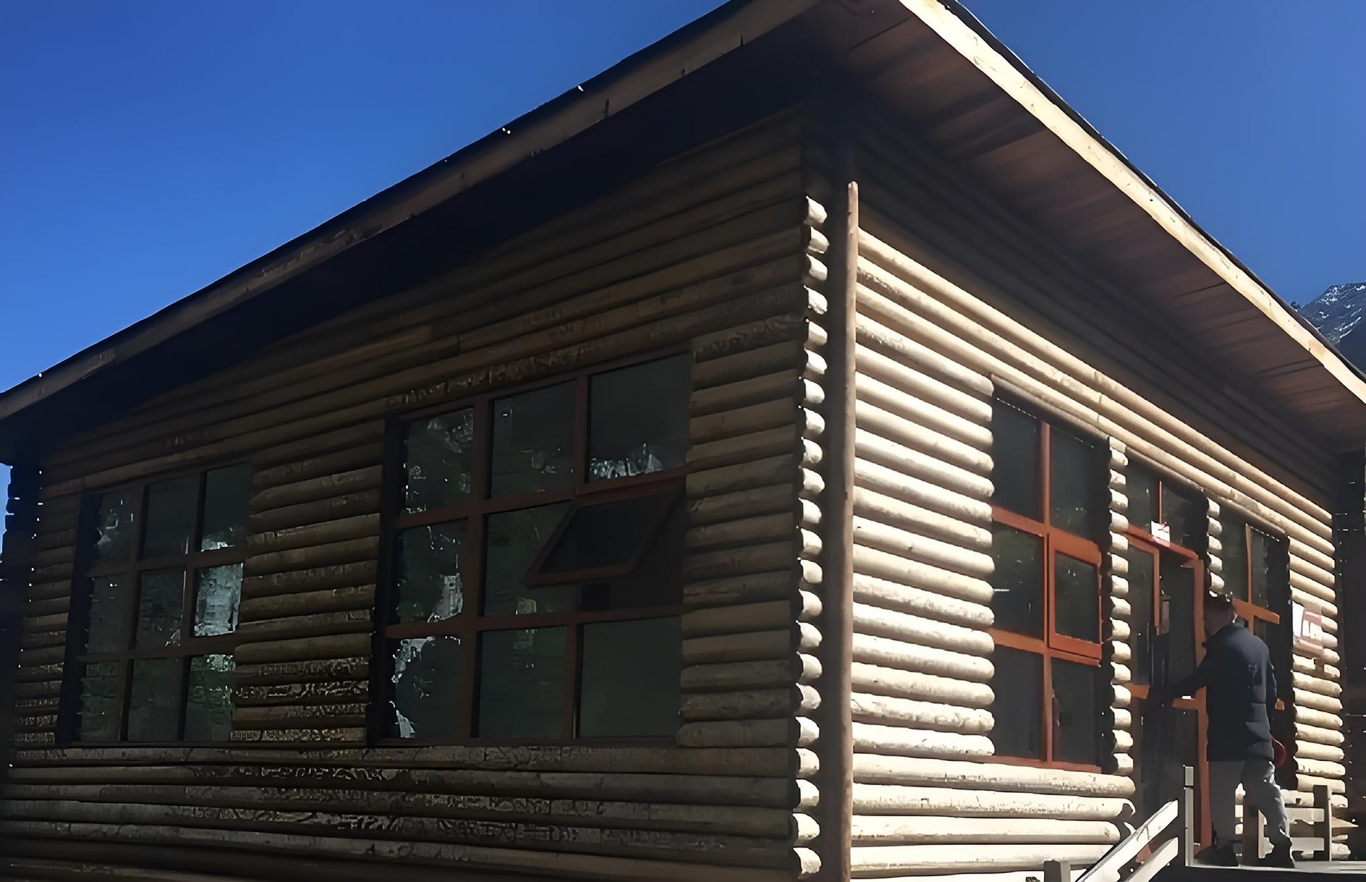
Single-storey LSF building.

A single-storey LSF building in Chengdu (China) is considered as a practical engineering application (Fig 15). The length and width of the building are both 8 m. Because the establishment of the thermal bridge is complex, the computational load of the software is very high. In addition, the mutual coupling between the thermal bridge junctions may affect the results of the calculation. Therefore, a novel calculation method is used instead of the transient simulation method. This method consists in calculating the energy consumption of the thermal bridge before and after improvement as:

$$Q=D\times K^{2D}\times b_{t}\times HDH18$$ (6)

where *Q* is the energy consumption of the thermal bridge during heating seasons (kW·h/a), *D* is the thermal bridge area (m^2^), *K*^*2D*^ is the two-dimensional heat transfer coefficient of the thermal bridge (W/(m^2^·K)), *b*_*t*_ is the temperature correction factor which is usually 1 above the ground and 0.5 below it, and HDH18 denotes the heating degree hours (34.437 kK·h/a) [37].

The results of the calculation are shown in Table 3. It can be seen that before improvement, the energy consumption of the thermal bridge at the external wall-beam junction is 22.63 kW·h and the energy consumption per unit area is 12.57 kW·h/m^2^. In addition, the energy consumption of the thermal bridge at the external corner of the external wall is 47.80 kW·h, and the energy consumption per unit area is 8.3 kW·h/m^2^. Moreover, the energy consumption of the thermal bridge at the cornice is 48.61 kW·h, and the energy consumption per unit area is 13.81 kW·h/m^2^. After improvement, the energy consumption of the thermal bridge at the external wall-beam junction is 11.78 kW·h/m^2^, which denotes a reduction of 10.85 kW·h. Furthermore, the energy consumption of the thermal bridge at the external corner of the external wall is 29.95 kW·h, which denotes a reduction of 17.85 kW·h, and that at the cornice is 35.03 kW·h, representing a reduction of 13.58 kW·h. This demonstrates that the thermal bridge can be significantly improved in order to decrease the heat transfer to the environment.

**Table 3:** Comparison between the energy consumption before and after improvement.

Thermal bridge name	Thermal bridge area (m^2^)	Before improvement		After improvement	
		Energy consumption (kW·h)	Energy consumption per unit area (kW·h/m^2^)	Energy consumption (kW·h)	Energy consumption per unit area (kW·h/m^2^)
Exterior wall -beam	1.80	22.63	12.57	11.78	6.54
External corner of external wall	5.76	47.80	8.30	29.95	5.20
Cornice	3.52	48.61	13.81	35.03	9.95

## 5. Conclusion

In this paper, the thermal bridge of external wall-beam junctions, external corner of external wall, internal corner of external wall, and cornice were first measured using an infrared thermal imager. The obtained results showed that the temperature at the thermal bridge parts significantly changes and the energy loss is large. The THERM software was then used to quantitatively analyze the temperature and heat flux distribution of the thermal bridge. Afterwards, the values of K^2D^ and Ψ of the thermal bridge were calculated. Finally, various methods were used for different thermal bridge parts to achieve the design standard of thermal bridge free, and make the building achieve an energy saving effect. The main results can be summarized as follows:

(1) For the thermal bridge of the external wall-beam junction, when the thickness of the RW thermal insulation layer becomes greater than 75 mm, the Ψ-value is less than 0.01 W/(m·K), which can meet the design standard of thermal bridge free.

(2) For the underlying conditions, XPS and EPS are used instead of RW as thermal insulation material of the external wall, which shows that the design standard of thermal bridge free cannot be met. When a PU thickness greater than 65 mm is used as thermal insulation material of the external wall of the external corner, the design standard of thermal bridge free is met. PU can replace RW for the internal corner of the external wall in order to meet the design standard of thermal bridge free.

(3) The method for reducing the thermal bridge of the cornice consists in adding a PU thermal insulation layer having a thickness greater than 20 mm at the inside room. This helpful to decrease the energy loss of the cornice, and then achieve the design standard of thermal bridge free.

In future work, renewable materials or bio-based materials will be used as thermal insulation materials of LSF to increase the thermal insulation performance and reduce the environmental damage and carbon emissions. In addition, it is necessary to increase the waterproof and fireproof performance of the thermal insulation materials. This will allow to meets the increasingly stringent building energy efficiency standards and environmental protection requirements. Therefore, further studying building thermal insulation materials for increasing their performance and sustainability allow to reach green buildings.

## References

[1] Research report of China buildings and urban infrastructure carbon emissions-2023. (In chinese).

[2] Santos P. Energy Efficiency of Lightweight Steel-Framed Buildings. 2017. Energy Efficient Buildings.

[3] Yu SS, Liu YF, Wang DJ, et al. (2021). Review of thermal and environmental performance of prefabricated buildings: Implications to emission reductions in China. Renewable & Sustainable Energy Reviews, 137,110472. doi: 10.1016/j.rser.2020.110472

[4] Xin Chen H.B. Blum, Krishanu Roy, et al. (2021). Cold-formed steel portal frame moment-resisting joints: Behaviour, capacity and design. Journal of Constructional Steel Research, 183,106718. doi: 10.1016/j.jcsr.2021.106718

[5] Chen Xin, Boston Megan, Bell Darrin. et al. (2023). Moment capacity of eaves brackets of cold-formed steel portal frames. Thin-Walled Structures, 189,110947. 10.1016/j.tws.2023.110947.

[6] Paul B., Roy K., Fang Z. et al. (2024). Moment Capacity of Bolted-Side Plate for the Eaves Joint of Nested Tapered Box Beam Portal Frame. International Journal of Steel Structures, 24(5),1189–1201. 10.1007/s13296-024-00888-7.

[7] Ilomets S, Kuusk K, Paap L, et al. (2017). Impact of linear thermal bridges on thermal transmittance of renovated apartment buildings. Journal of Civil Engineering and Management,23(1),96–104.

[8] Erhorn-Kluttig H H E. (2009). Impact of thermal bridges on the energy performance of buildings. ASIEPI Project,3,148.

[9] Liang H, Krishanu Roy, Fang ZY, et al.(2022). A Critical Review on Optimization of Cold-Formed Steel Members for Better Structural and Thermal Performances. Buildings, 12,34. 10.3390/buildings12010034.

[10] Yazeed Al-Radhi Krishanu Roy, Liang Hao et al. (2023).Thermal performance of different construction materials used in New Zealand dwellings comparatively to international practice–A systematic literature review. Journal of Building Engineering, 72, 106346. 10.1016/j.jobe.2023.106346.

[11] Pérez-Carraminana C, Morena-Marqués A, Gonzalez-Avilés ÁB, et al. (2024). Influence of Balcony Thermal Bridges on Energy Efficiency of Dwellings in a Warm Semi-Arid Dry Mediterranean Climate. Buildings,14,703. 10.3390/buildings14030703.

[12] Zhao K, Jiang Z, Huang Y, et al. (2022). The method of reducing energy loss from thermal bridges in residential buildings with internal insulation in the hot summer and cold winter zone of China. Journal of Building Engineering, 62,105421. 10.1016/j.jobe.2022.105421.

[13] De Angelis E E S. (2014).Light steel-frame walls: Thermal insulation performances and thermal bridges. Energy Procedia,45,362–371.

[14] Zalewski L, Lassue S, Rousse D, et al. (2010). Experimental and numerical characterization of thermal bridges in prefabricated building walls. Energy Conversion and Management,51(12),2869–2877.

[15] Ge H, Baba F. (2015). Dynamic effect of thermal bridges on the energy performance of a low-rise residential building. Energy and Buildings,105,106–118. 10.1016/j.enbuild.2015.07.023.

[16] Baba F, Ge H. (2016). Dynamic effect of balcony thermal bridges on the energy performance of a high-rise residential building in Canada. Energy and Buildings,116,78–88. 10.1016/j.enbuild.2015.12.044.

[17] Ge H, Baba F. (2017). Effect of dynamic modeling of thermal bridges on the energy performance of residential buildings with high thermal mass for cold climates. Sustainable Cities and Society,34,250–63. 10.1016/j.scs.2017.06.016.

[18] Song JH, Lim JH, Song SY. (2016). Evaluation of alternatives for reducing thermal bridges in metal panel curtain wall systems. Energy and Buildings,127,138–158. 10.1016/j.enbuild.2016.05.078.

[19] O’Grady M, Lechowska AA, Harte AM. (2017). Quantification of heat losses through building envelope thermal bridges influenced by wind velocity using the outdoor infrared thermography technique. Applied Energy,208,1038–1052. 10.1016/j.apenergy.2017.09.047.

[20] O’Grady M, Lechowska AA, Harte AM. (2017). Infrared thermography technique as an in-situ method of assessing heat loss through thermal bridging. Energy and Buildings,135,20–32. 10.1016/j.enbuild.2016.11.039.

[21] Garrido I, Laguela S, Arias P, et al. (2018). Thermal-based analysis for the automatic detection and characterization of thermal bridges in buildings. Energy and Buildings,158,1358–1367. 10.1016/j.enbuild.2017.11.031

[22] Mao S, Kan A, Wang N. (2020). Numerical analysis and experimental investigation on thermal bridge effect of vacuum insulation panel. Applied Thermal Engineering,169,114980. 10.1016/j.applthermaleng.2020.114980.

[23] Milovanović B, Bagarić M, Gaši M, et al. (2022). Case Study in Modular Lightweight Steel Frame Construction: Thermal Bridges and Energy Performance Assessment. Applied Sciences-Basel,12(20),10551. 10.3390/app122010551.

[24] Kim CM, Choi JS, Jang H, et al. (2021). Automatic Detection of Linear Thermal Bridges from Infrared Thermal Images Using Neural Network. Applied Sciences-Basel,11(3),931. 10.3390/app11030931.

[25] Romero MJ, Aguilar F, Vicente PG. (2021). Analysis of design improvements for thermal bridges formed by double-brick facades and intermediate slabs for nZEB residential buildings in Spain. Journal of Building Engineering, 44,103270. 10.1016/j.jobe.2021.103270.

[26] Zhang X, Jung GJ, Rhee KN. (2022). Performance Evaluation of Thermal Bridge Reduction Method for Balcony in Apartment Buildings. Buildings,12(1),63. 10.3390/buildings12010063.

[27] Real S, Gloria Gomes M, Moret Rodrigues A, et al. (2016). Contribution of structural lightweight aggregate concrete to the reduction of thermal bridging effect in buildings. Construction and Building Materials,121,460–470. 10.1016/j.conbuildmat.2016.06.018.

[28] Miguel A. Paya-Marin, Krishanu Roy, Jian-Fei Chen et al. (2020). Large-scale experiment of a novel non-domestic building using BPSC systems for energy saving. Renewable Energy,152,799–811. 10.1016/j.renene.2020.01.100.

[29] Tong Zhu, An QS Liu XH, et al. Heat transfer (The 7th edition). Beijing: China Architecture & Building Press [M]. (2020) (in Chinese).

[30] Ministry of Housing and Urban Rural Development of the People’s Republic of China. Thermal design code for civil building (GB50176-2016). Beijing: China Architecture & Building Press. 2016. (In chinese).

[31] Berardi U, Naldi M. (2017). The impact of the temperature dependent thermal conductivity of insulating materials on the effective building envelope performance. Energy and Buildings, 144,262–275. 10.1016/j.enbuild.2017.03.052.

[32] Roque Eduardo, Santos P. (2017).The effectiveness of thermal insulation in lightweight steel-framed walls with respect to Its position. Buildings,7,13.

[33] Jb Yang. Analysis and Improvement of Thermal Bridge of Building Envelope Based on a Prefabricated Building Changsha. Changsha:Hunan University.2016. (In chinese).

[34] Kaufmann B, Feist W. Design and Construction Guidelines for Passive Houses in Germany. Beijing: China Architecture & Building Press. 2015. (In chinese).

[35] Kapoor Divyansh R., Peterman Kara D. (2021). Quantification and prediction of the thermal performance of cold-formed steel wall assemblies. Structures, 30,305–315. doi: 10.1016/j.istruc.2020.12.060

[36] Zhang M. (2024). Research progress and application of building exterior wall materials under the perspective of energy saving and thermal insulation. Guangdong Building Materials, 10, 181–183(In chinese).

[37] Tsinghua University. DeST-h software. 2014.

